# Cui Bono?

**Published:** 1850-01

**Authors:** 


### THE WESTERN JOURNAL.

THIRD SERIES.—VOL. V.—NO. I.

LOUISVILLE, JANUARY 1, 1850.

### CUI BONO?

The readers of the November number of this Journal will remember that we made some editorial remarks on the subject of the surgical papers of Professor B. W. Dudley. The spirit of those remarks has been misconstrued in the December number of the Transylvania Journal of Medicine, and this misconstruction is made the basis of a violent personal attack upon one of the editors of this Journal. We shall imitate neither the spirit, temper, taste, nor language of this singular article, but we owe it to ourself to say something on the misrepresentation to which we have been subjected. The article to which we allude, represents that we have done some wrong or injustice to Professor B. W. Dudley, but we hold that we are as incapable of doing anything of the kind, as the editor of the Transylvania Journal can be, and this opinion is founded on years of admiration of, and affection for that distinguished personage as a surgeon, teacher, and gentleman. We have no motive to do him wrong, and it would require a more powerful one than we have ever yet felt, to induce such an act. The feelings of veneration, gratitude, and esteem we formed for the Professor many years ago, founded on an intimate knowledge of his character, have undergone no change, and we have some hope that they are immutable.—But it is due to freedom of opinion, to liberty of speech itself, to say that we are not aware of anything, in the way of position, eminence, age or talent, that places a man beyond the reach of fair and manly criticism, especially when personal regard guides the pen. In the article which has aroused the temper of the Transylvania Journal, we thought we did justice to the eminent character of Professor Dudley as a teacher and surgeon, and did not for a moment suppose that we were committing a heinous offence. What we had to say was expressed in anythiug but offensive terms; we intended to be decorous and respectful, and we are well satisfied that we did not pass beyond the limits of our intentions.

There was, in our estimation, an indifferent foundation for the assault made upon us, and self-respect prompts us to be careful that we do not build a similar superstructuré on a similar basis. We are certain that we do not misapprehend the tastes or interests of the subscribers to this Journal, in supposing that they would be disgusted with personal maledictions, and squabbling on the pages of a work devoted to medical science.

We had hoped for a pleasant intercourse with the Transylvania Journal; we hoped that differences of judgment and opinion would be expressed in terms of professional courtesy, and that the only competition between the two Journals would be found in an endeavor to advance medical science. These hopes have been dissipated, and as we have no kind of desire to maintain such an intercourse as the Transylvania Journal has indicated, we must decline for the future any connection with that Journal.

